# Etymologia: Hemagglutinin and Neuraminidase

**DOI:** 10.3201/eid2410.ET2410

**Published:** 2018-10

**Authors:** Ronnie Henry, Frederick A. Murphy

**Keywords:** hemagglutinin, neuraminidase, influenza, influenza virus, viruses, glycoprotein, subtypes, red blood cells, erythrocytes, agglutination, sialic acid, sialidase, neuraminic acid, George K. Hirst, Alfred Gottschalk

## Hemagglutinin [heʹmə-glooʹtĭ-nin] and neuraminidase [noorʹə-minʹĭ-dās]

In 1941, virologist George K. Hirst discovered that adding influenza virus to red blood cells (erythrocytes) in a test tube caused the cells to agglutinate, mediated by one of the virus surface glycoproteins, hemagglutinin (from the Greek *haima*, “blood,” + Latin *gluten*, “glue”) (Figure). Alfred Gottschalk later showed that hemagglutinin binds virus to host cells by attaching to sialic acids (from the Greek *sialon*, “saliva”) on carbohydrate side chains of cell-surface glycoproteins and glycolipids. The other influenza virus surface protein, neuraminidase (referring to brain lipids from which it was first derived) is a virus receptor-destroying enzyme that removes its substrate, sialic acids, from infected cell surfaces so that newly made progeny viruses are released to infect additional cells. At present, 18 hemagglutinin subtypes (H1–H18) and 11 neuraminidase subtypes (N1–N11) are recognized.

**Figure:**
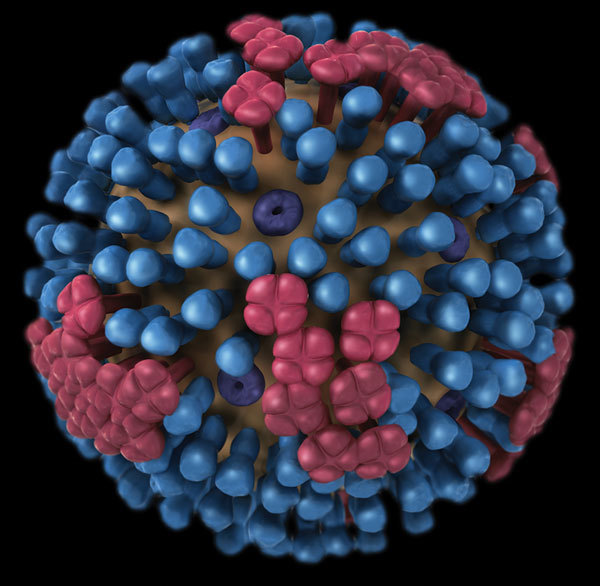
Image of influenza virus showing hemagglutinin (blue) and neuraminidase (red) proteins on the surface of the virus. Content source: Centers for Disease Control and Prevention, National Center for Immunization and Respiratory Diseases (NCIRD).

## References

[1] Gottschalk A. The chemistry and biology of sialic acids and related substances. London: Cambridge University Press; 1960.

[2] Hirst GK. The agglutination of red cells by allantoic fluid of chick embryos infected with influenza virus. Science. 1941;94:22–3. 10.1126/science.94.2427.22 17777315

[3] Hirst GK. Adsorption of influenza hemagglutinins and virus by red blood cells. J Exp Med. 1942;76:195–209. 10.1084/jem.76.2.195 19871229 PMC2135226

